# Bone cement spacer: a novel technique for treating a complicated case of developmental dysplasia of the hip with an injured acetabulum: Case report with 7-year follow-up

**DOI:** 10.1097/MD.0000000000018655

**Published:** 2020-01-03

**Authors:** Abdulmonem Alsiddiky, Raheef Alatassi, Saud Alfayez, Fahad Alhuzaimi, Mahdi Alqarni

**Affiliations:** a King Saud University, College of Medicine, Research Chair of Spinal Deformities, Department of Orthopedics; b Security Forces Hospital, Department of Orthopedic Surgery, Riyadh, Saudi Arabia.

**Keywords:** acetabuloplasty, bone cements, developmental dysplasia of the hip, hip dislocation, spacer

## Abstract

**Rationale:**

Developmental dysplasia of the hip (DDH) has an incidence of 5 per 1000 newborns and its management depends on various factors. We present a rare case of DDH with soft tissue obliteration and a bony prominence in the center of the acetabulum after failed open reduction and acetabuloplasty.

**Patient concerns:**

A 20-month-old girl presented to our clinic with right hip stiffness after undergoing open reduction and acetabuloplasty at another hospital.

**Diagnoses:**

The diagnosis of DDH was made using a computed tomography scan that revealed a right hip dislocation with soft tissue obliteration and a bony prominence in the center of the acetabulum.

**Interventions:**

We used a novel technique for treating the rare presentation of complicated DDH with massive soft tissue obliteration and bony prominence in the center of the acetabulum after failed open reduction and acetabuloplasty. The right hip was surgically explored. The acetabulum was deepened and resurfaced. Bone cement was applied over the acetabulum to prevent future ankylosis.

**Outcomes:**

At the follow-up 7 years after the last surgery, the patient had regained full range of motion and a properly reduced right hip with optimal acetabular coverage on radiographs.

**Lessons:**

Care must be taken in any patient with DDH who presents with hip redislocation after open reduction. If deepening and resurfacing of the acetabulum are required, bone cement could be used as a temporary spacer for 8 weeks; this was key in treating our patient.

## 1 Introduction

Developmental dysplasia of the hip (DDH) describes a spectrum of hip abnormalities ranging from mild dysplasia of the acetabulum to complete dislocation of the hip joint. An incidence rate of 5 per 1000 newborns has recently been reported using ultrasound at the age of 3 months.^[1]^ Late presentation of DDH ranges from 0.07 to 2.0 cases per 1000 births.^[2]^

The management of DDH depends on various factors, including the patient's age upon presentation. In infants up to 6 months old, DDH can be managed conservatively with a Pavlik harness. However, children older than 2 years will likely require extensive surgery with open reduction and pelvic and potentially femoral shortening osteotomy. Patients with DDH must be appropriately assessed and managed to avoid complications that severely compromise hip function.^[3–5]^

We present a rare case of DDH with soft tissue obliteration and a bony prominence in the center of the acetabulum after failed open reduction and acetabuloplasty. Given the unusual presentation, we designed a novel treatment strategy that entailed the excision of the soft tissue and bony prominence, deepening of the floor of the acetabulum, and temporary insertion of a molded bone-cement spacer.

This case report was written according to the recently published CARE criteria as it is used for supporting transparency and accuracy in publication of case reports.

## 2 Case presentation

**Figure 1:**
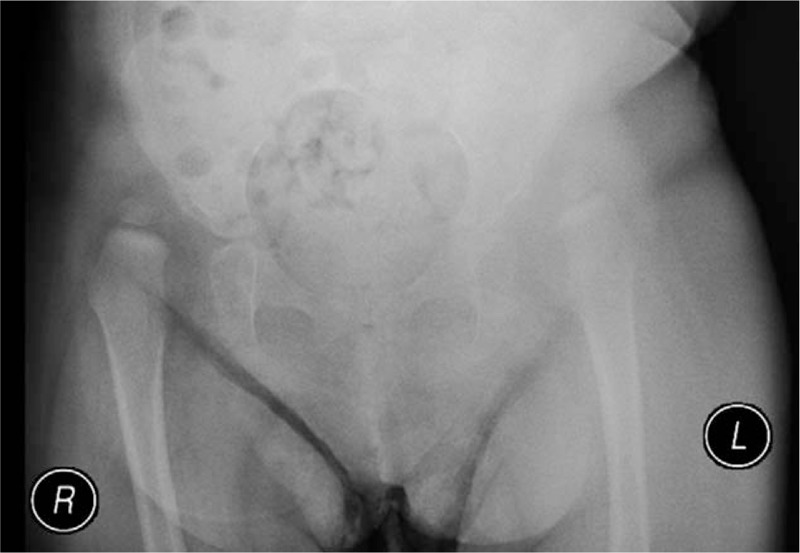
Fourteen-month-old girl with bilateral developmental dysplasia of the hips: Anteroposterior radiograph of the pelvis showing dislocation of both hips.

A 14-month-old girl presented to a private hospital with bilateral DDH. Plain radiographs showed a complete dislocation of both hips (Fig. 1). She underwent staged open hip reduction and acetabuloplasty. The left hip was treated first, followed by the application of a hip spica cast for 6 weeks. The right hip was operated afterward, followed by immobilization in a hip spica for 6 weeks and then in a broomstick cast for another 6 weeks. After the removal of the cast, the right hip was dislocated again. The patient was treated with closed reduction under anesthesia and immobilized in a hip spica for another 2 months. Upon removal of the cast, the right hip had dislocated again, and there was stiffness in both hips.

**Figure 2:**
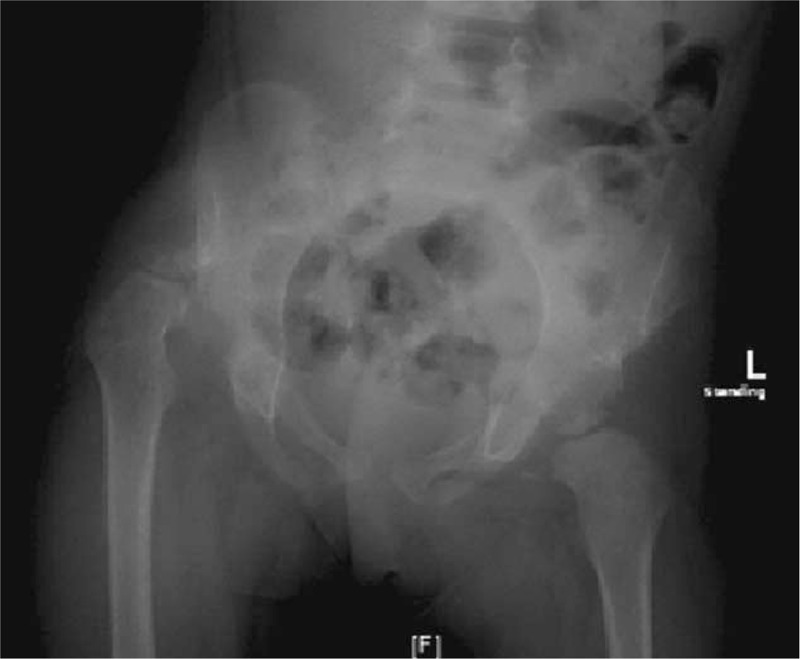
Twenty-month-old girl with bilateral developmental dysplasia of the hips: Anteroposterior radiograph of the pelvis showing complete dislocation of the right hip.

At the age of 20 months, the patient was referred to our hospital with severe stiffness of both hips and a dislocated right hip (Fig. 2). Because of the stiffness, we referred her to the physiotherapist to improve the range of motion (ROM) in both hips. Four months later, the ROM was acceptable (Table 1).

**Table 1:**
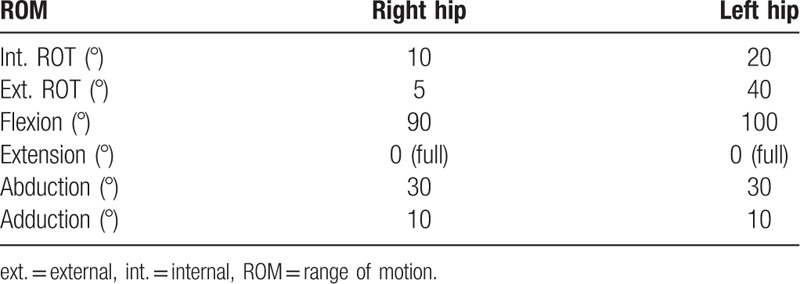
Range of motion of both hips after physiotherapy.

**Figure 3:**
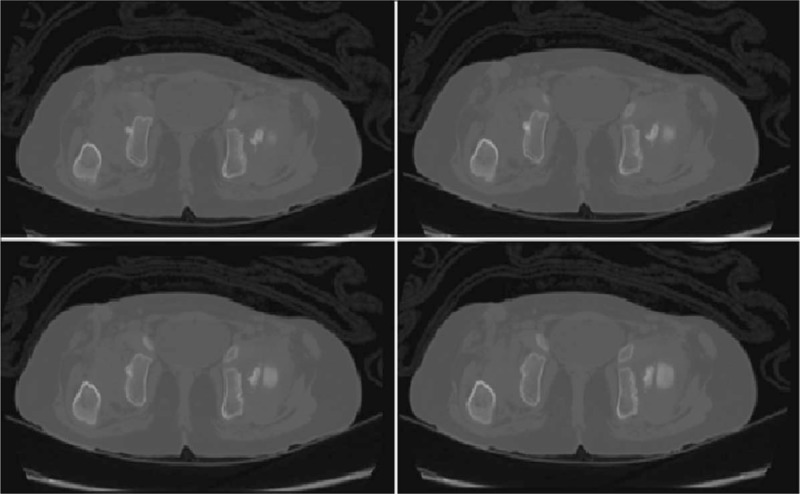
Twenty-month-old girl with bilateral developmental dysplasia of the hips: Multiple axial views of a computed tomography scan revealing a right obliterated acetabulum with a central bony prominence.

A computed tomography (CT) scan revealed a central acetabular bony prominence posterior to the triradiate cartilage that measured around 1.5 cm in width at its base and had an 8-mm long tooth-like end projecting into the joint. The femoral head was dislocated in the posterior-lateral direction (Fig. 3). The parents were informed about the nature of their daughter's condition and the purpose of performing our novel operation, which, to our knowledge, has never been performed before, and they provided consent to perform our technique and for publication of the case.

**Figure 4:**
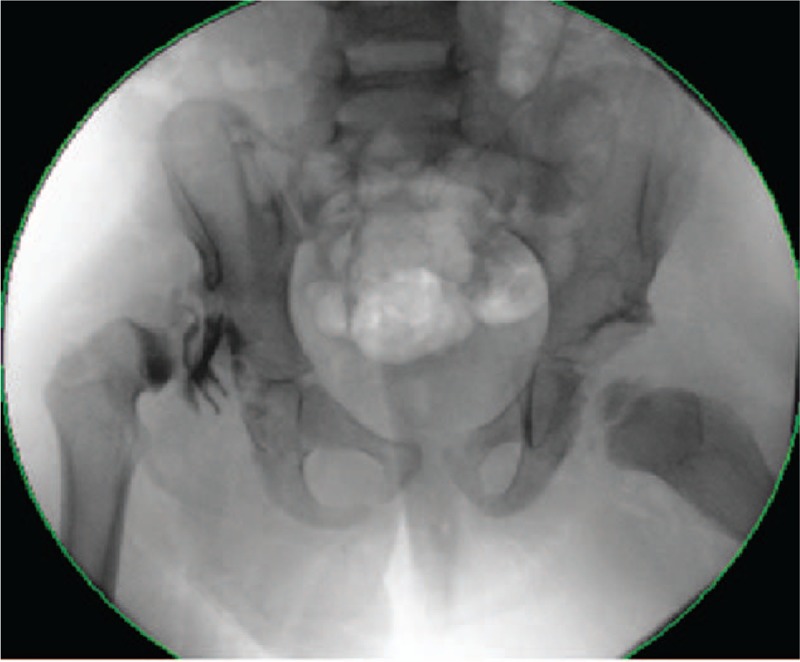
Twenty-month-old girl with bilateral developmental dysplasia of the hips: Intraoperative arthrogram showing a filling defect of the right hip.

Under general anesthesia, examination and arthrography of the right hip were performed and revealed an obliterated right acetabulum (Fig. 4). We accessed the joint through the previous incision and excised all fibro-fatty tissue obliterating the acetabulum. Consecutively, we removed the bony prominence with its central spur and deepened the floor of the acetabulum using a dental bur to accommodate the head of the femur.

**Figure 5:**
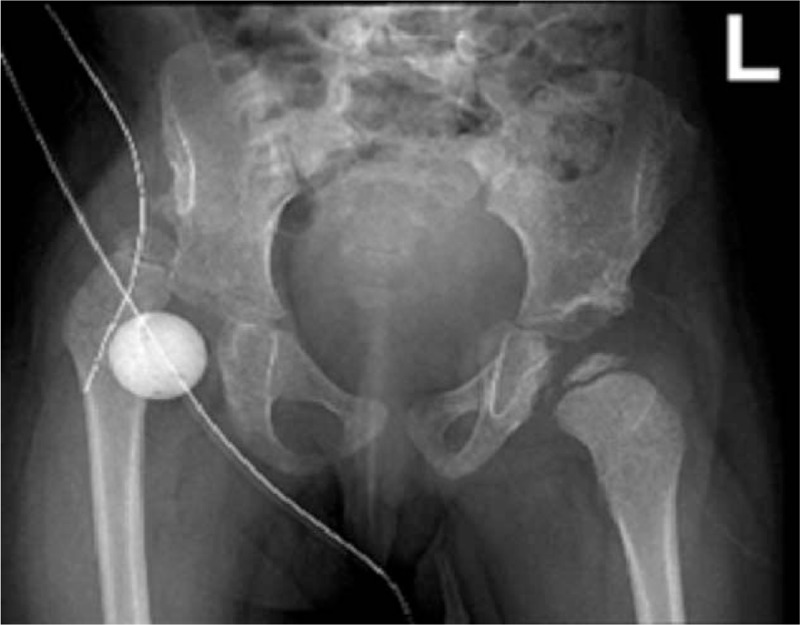
Twenty-month-old girl with bilateral developmental dysplasia of the hips: Postoperative anterio-posterior image after placement of a ball-shaped bone cement spacer and reduction of the right hip.

We decided to insert a spacer made of bone cement inside the acetabular cavity to prevent ankylosis. We formed the cement into a ball about the same size and shape of the head of the femur. We secured the position of the cement ball in the acetabulum with multiple non-absorbable sutures to ensure the healing of the acetabular surface (Fig. 5).

A hip spica cast was applied for 2 months to allow the acetabular bone to heal and to allow fibrocartilage to cover the bone so that the smoothened surface was ready to accommodate the head of the femur.

**Figure 6:**
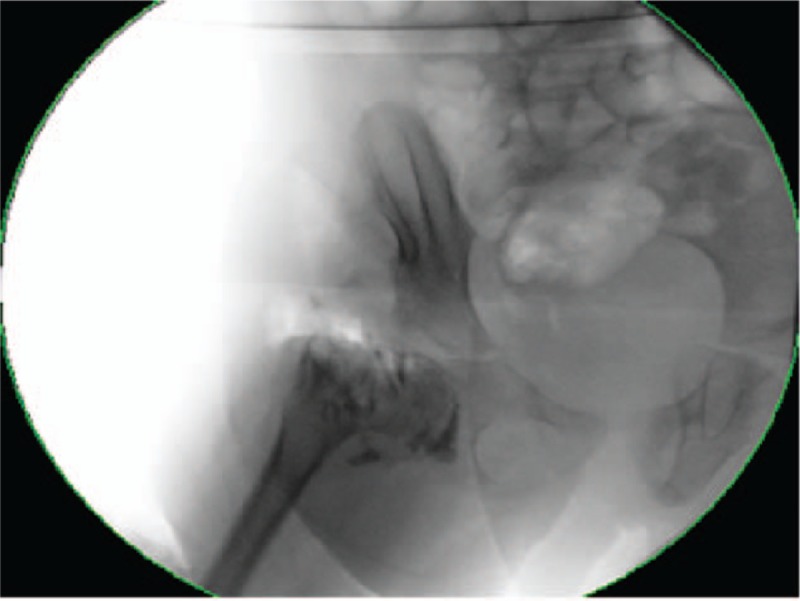
Twenty-two-month-old girl with bilateral developmental dysplasia of the hips: Arthrogram after bone cement removal 8 weeks after its insertion demonstrating the ability to reduce the right hip properly.

Eight weeks later, we removed the spacer and explored the acetabulum intraoperatively. We found that it was healed completely and covered with fibrocartilage of an optimal depth and congruency to accommodate the femoral head. The hip was reduced, and a capsulorrhaphy was performed. A hip spica cast was applied for 4 weeks, followed by a broomstick cast for another 4 weeks. After removing the cast, arthrography of the hip revealed a concentrically reduced hip with acceptable coverage of the femoral head (Fig. 6).

**Figure 7:**
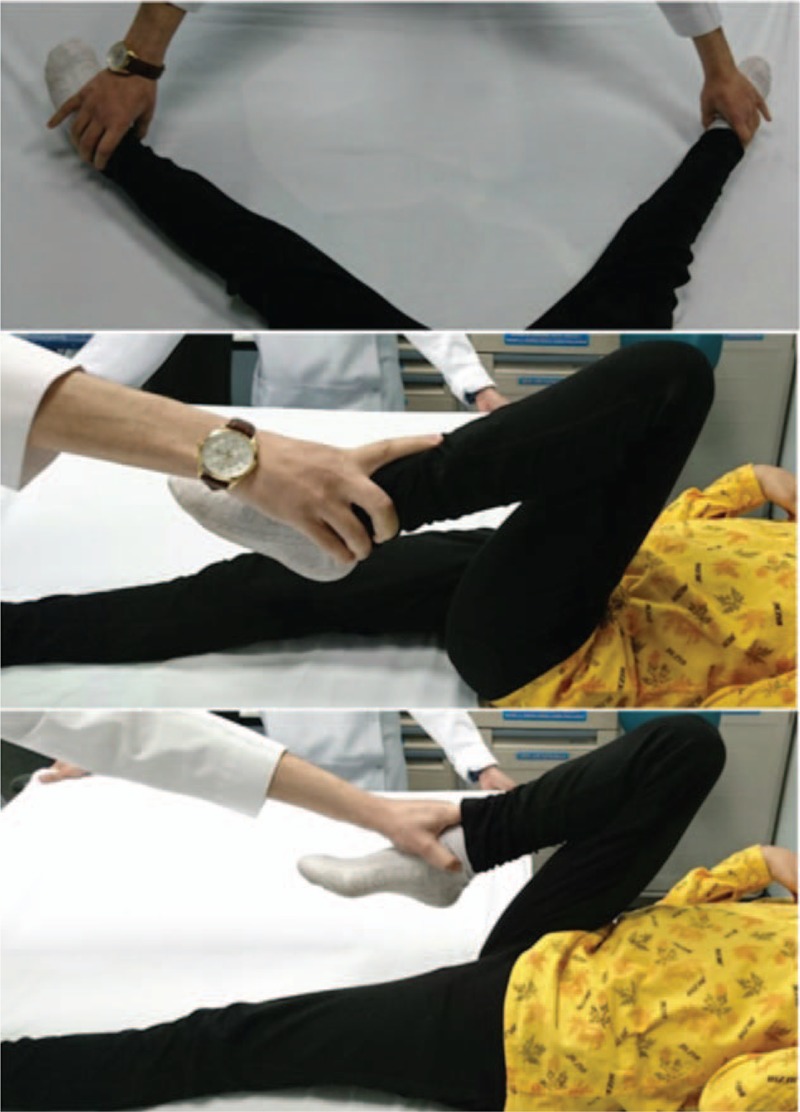
Patient with bilateral developmental dysplasia of the hip 7 years after novel treatment of the right hip: Active range of motion when flexing and abducting the hips.

**Figure 8:**
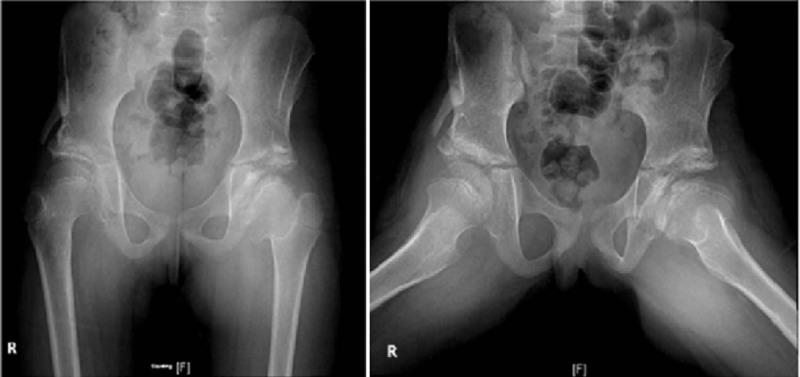
Patient with bilateral developmental dysplasia of the hip 7 years after novel treatment of the right hip: (A) Anteroposterior radiograph of the pelvis and (B) lateral frog view radiograph showing proper reduction and acetabular coverage.

Mild stiffness was noticed in both hips postoperatively, and we referred the patient again to the physiotherapist for ROM exercises. During the follow-up, a significant improvement in her gait and the ROM of both hips was noticed. Seven years after the surgery, she had almost full ROM of both hips with normal gait (Table 2). She could cross her legs and perform daily activities without any restrictions (Fig. 7). The radiographic assessment showed concentrically reduced hips with good acetabular coverage and no ankylosis (Fig. 8).

**Table 2:**
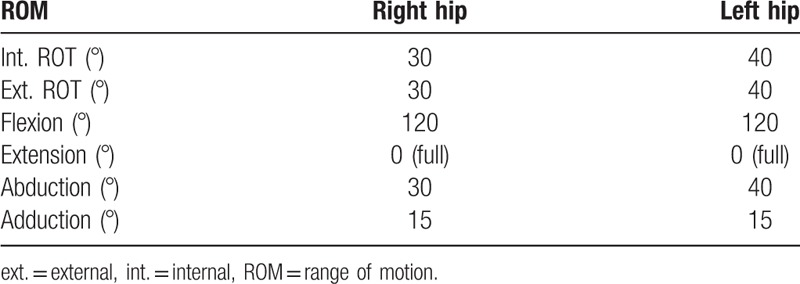
Range of motion of both hips after physiotherapy at the 7-year follow-up.

## 3 Discussion

This rare DDH case presented a challenge because of the bony prominence in the acetabulum and the stiffness of the dislocated hip. Our novel technique was conceived to address and manage these orthopedic problems and achieve the desired outcomes of proper reduction and optimal function of the hip.

Stiffness after joint immobilization is common, and physiotherapy is generally recommended after removing a hip spica cast.^[3,6]^ It is crucial to restore the ROM through physiotherapy before any surgical intervention. Further, physiotherapy should also be performed postoperatively if the ROM of the hip joint is not optimal.

We believe that the most challenging part in such cases is to identify and manage the damaged acetabulum and its central bony prominence. Therefore, we further recommend CT scans or magnetic resonance imaging in all patients with a redislocated hip after open reduction to meticulously assess the femoral head and acetabulum for any abnormalities.^[7]^

In our case, the decision to deepen and resurface the acetabulum to create an optimal socket for the femoral head was made once we realized the excessive thickness of the acetabulum and the bony acetabular prominence. We believe this prominence could have been caused by aggressive resection of the acetabular pulvinar or a direct injury to the acetabulum during the initial surgery. However, ankylosis of the hip joint becomes highly likely in such cases because the resurfaced acetabular bone is prone to fuse with the femoral head. This may lead to a catastrophic functional outcome, especially since the patient was already 2 years old and had suboptimal function of the contralateral hip.^[8,9]^

A further challenge is the small size of the acetabulum in children, which hinders the deepening of its floor and removal of any bone formations. Using the dental bur, which has a smaller diameter than the orthopedic bur, we overcame this challenge. The acetabulum was resurfaced to its optimal shape and size while minimizing any damage and preserving the cartilage as much as possible.

We decided to repurpose bone cement, usually used in joint arthroplasty, as a spacer. The benefits of the cement were the low risk of allergic reactions, as cement is normally used in the joint, and the ability to form the cement in the way that we wanted. The size and shape of both the acetabulum and the femoral head differ between patients, and cement allows the creation of a spacer specific to a patient's anatomy. Another advantage of cement is that, once it hardens, its surface will be smooth, thus not causing injury to the cartilage of the acetabulum and being easy to remove.

The spacer should be left inside the joint for at least 8 weeks so that the fibrous tissue can form over the resected side of the acetabulum to prevent ankylosis. It has been shown that after the primary reduction of the dislocated femoral head, the acetabulum joint space will fill with hypertrophied acetabular cartilage and/or fibrous tissues.^[10,11]^ After removing the bone cement, it should be inspected to confirm that fibrous cartilage has indeed formed before reducing the femoral head. Then, a hip spica cast should be applied for at least 4 weeks to maintain the reduction.

In conclusion, we described a new technique to manage a rare complication of failed open reduction and acetabuloplasty in DDH. Careful clinical and radiological assessment is required in any DDH patient with redislocation after open reduction. Hip stiffness should be addressed before any surgical intervention. After deepening and resurfacing of the acetabulum, bone cement was used as a spacer for 8 weeks to allow fibrocartilage to form. The bone cement spacer is safe, easy to use, and affordable. After removal of the bone cement, the hip was reduced, and a hip spica applied to maintain the reduction. The patient is free of complaints with almost full ROM 7 years after the intervention.

## Acknowledgments

The authors sincerely thank King Saud University, Vice Deanship of Research Chairs and Research Chair of Spinal Deformities for their enthusiastic assistance.

## Author contributions

**Conceptualization:** Abdulmonem Alsiddiky.

**Data curation:** Raheef Alatassi.

**Project administration:** Raheef Alatassi.

**Resources:** Raheef Alatassi.

**Supervision:** Abdulmonem Alsiddiky.

**Visualization:** Fahad Alhuzaimi.

**Writing – original draft:** Raheef Alatassi.

**Writing – review & editing:** Abdulmonem Alsiddiky, Raheef Alatassi, Saud Alfayez, Fahad Alhuzaimi, Mahdi Alqarni.

Raheef Alatassi orcid: 0000-0003-0822-1118.
